# Predicting health related deprivation using loyalty card digital footprints

**DOI:** 10.23889/ijpds.v8i3.2286

**Published:** 2023-09-11

**Authors:** Gavin Long, Georgiana Nica-Avram, Anya Skatova, Ross Otto

**Affiliations:** 1 Department of Psychology, McGill University; 2 Nottingham Business School, University of Nottingham; 3 Bristol Medical School, University of Bristol

## Introduction & Background

A key premise of rational choice prescribes that decision-makers ought to ignore irrelevant, inferior alternative options. Consider for example the choice between two wines, where the value of an option is computed across two dimensions: quality and price. When deliberating about which wine to choose, one’s propensity to choose between two otherwise equally preferred wines should be not influenced by the introduction of a third clearly inferior option (being both of lower quality and more expensive than one of the original alternatives). Yet, a large body of work suggests that both people and animals routinely violate this premise in their decisions—in laboratory experiments, the introduction of irrelevant “decoys” into a choice set biases decision-making. However, these decoy effects are less understood in large-scale contexts of real-world decision-making, where choice sets can be large, and preference informed by consumers’ histories of experience.

## Objectives & Approach

We explored whether the presence of irrelevant, “decoy” alternative options influenced wine purchases in a large real-world dataset of UK wine purchases. From shopping transaction records, we extracted all red and white wine purchases over a one month period. Our analyses examined 3.6M wine purchases made by 755,158 unique customers.

## Relevance to Digital Footprints

We deployed shopping history data which is a popular example for digital footprints allowing us to track people’s choices and decisions over long periods of time.

## Results

We find that among pairs of wines that appear across many different contexts (i.e., stores with different product assortments) and trade off on quality and price, the presence of decoy options— similar options that were dominated by the focal option—made consumers more likely to purchase the focal option (a hallmark of the “attraction effect”). Furthermore, we find that sensitivity to this effect depended on consumers’ history of experience with the product, such that frequent shoppers were less likely to be sensitive to decoy effects in their purchase behaviour.

## Conclusions & Implications

We examined whether real-world consumer decisions, evidenced in a large dataset of wine purchases in the United Kingdom, were subject to a canonical bias in multiattribute choice: the attraction effect. We found that wine purchases were systematically biased in favour of wines that dominated choice sets—a bias which was not observed when choice sets were not dominated. Together, these results extend laboratory-based accounts of decoy effects to real-world choices, and demonstrate how digital footprints data analysis can be linked to health, especially in terms of decision making which is associated with negative health outcomes.

